# Tailoring of TiAl6V4 Surface Nanostructure for Enhanced In Vitro Osteoblast Response via Gas/Solid (Non-Line-of-Sight) Oxidation/Reduction Reactions

**DOI:** 10.3390/biomimetics7030117

**Published:** 2022-08-25

**Authors:** Naotaka Ogura, Michael B. Berger, Pavan Srivas, Sunghwan Hwang, Jiaqi Li, David Joshua Cohen, Zvi Schwartz, Barbara D. Boyan, Kenneth H. Sandhage

**Affiliations:** 1 School of Materials Engineering, Purdue University, W. Lafayette, IN 47907, USA; 2 Department of Biomedical Engineering, Virginia Commonwealth University, Richmond, VA 23284, USA

**Keywords:** titanium alloy, implants, surface modification, nanostructure, non-line-of-sight, oxidation, calciothermic reduction, biomaterials, orthopaedic, dental

## Abstract

An aging global population is accelerating the need for better, longer-lasting orthopaedic and dental implants. Additive manufacturing can provide patient-specific, titanium-alloy-based implants with tailored, three-dimensional, bone-like architecture. Studies using two-dimensional substrates have demonstrated that osteoblastic differentiation of bone marrow stromal cells (MSCs) is enhanced on surfaces possessing hierarchical macro/micro/nano-scale roughness that mimics the topography of osteoclast resorption pits on the bone surface. Conventional machined implants with these surfaces exhibit successful osseointegration, but the complex architectures produced by 3D printing make consistent nanoscale surface texturing difficult to achieve, and current line-of-sight methods used to roughen titanium alloy surfaces cannot reach all internal surfaces. Here, we demonstrate a new, non-line-of-sight, gas/solid-reaction-based process capable of generating well-controlled nanotopographies on all open (gas-exposed) surfaces of titanium alloy implants. Dense 3D-printed titanium-aluminum-vanadium (TiAl6V4) substrates were used to evaluate the evolution of surface nanostructure for development of this process. Substrates were either polished to be smooth (for easier evaluation of surface nanostructure evolution) or grit-blasted and acid-etched to present a microrough biomimetic topography. An ultrathin (90 ± 16 nm) conformal, titania-based surface layer was first formed by thermal oxidation (600 °C, 6 h, air). A calciothermic reduction (CaR) reaction (700 °C, 1 h) was then used to convert the surface titania (TiO_2_) into thin layers of calcia (CaO, 77 ± 16 nm) and titanium (Ti, 51 ± 20 nm). Selective dissolution of the CaO layer (3 M acetic acid, 40 min) then yielded a thin nanoporous/nanorough Ti-based surface layer. The changes in surface nanostructure/chemistry after each step were confirmed by scanning and transmission electron microscopies with energy-dispersive X-ray analysis, X-ray diffraction, selected area electron diffraction, atomic force microscopy, and mass change analyses. In vitro studies indicated that human MSCs on CaR-modified microrough surfaces exhibited increased protein expression associated with osteoblast differentiation and promoted osteogenesis compared to unmodified microrough surfaces (increases of 387% in osteopontin, 210% in osteocalcin, 282% in bone morphogenic protein 2, 150% in bone morphogenic protein 4, 265% in osteoprotegerin, and 191% in vascular endothelial growth factor). This work suggests that this CaR-based technique can provide biomimetic topography on all biologically facing surfaces of complex, porous, additively manufactured TiAl6V4 implants.

## 1. Introduction

The attractive mechanical and chemical properties, as well as biocompatibility, of titanium (Ti) and Ti-based alloys have led to the extensive use of these materials in orthopaedic and dental implants [1,2,3]. Inspired by the hierarchical (micro-to-nanoscale) surface roughness of resorption pits formed on bone surfaces by osteoclasts prior to new bone formation by osteoblast progenitor cells, previous investigations have revealed that the surface topography of Ti-based implants, at both the micrometre scale (≥10^3^ nm) and nanometre scale (≤10^2^ nm), regulates osteoblastic cell behaviour to support osseointegration [4,5]. Bone marrow stromal cells (MSCs) cultured on these tailored surfaces increase osteogenic protein production, and net bone apposition and osseointegration are increased in vivo [6,7,8]. Whereas a variety of physical and chemical coating routes (e.g., ion-beam sputtering, magnetron sputtering, plasma spraying, RF sputtering, pulsed laser deposition, use of self-assembled monolayers [9,10,11,12,13,14,15]) have been examined for modifying the surfaces of Ti-based implants, the most common, clinically accepted method for creating micro-/meso-/nanoscale roughness on Ti-based implant surfaces is sandblasting with large grit followed by acid-etching (SLA process) [16,17].

Additive manufacturing (AM) to fabricate Ti-based orthopaedic implants for functional load-bearing applications has received increasing interest in recent years [18,19]. The advantages of AM include the direct fabrication of complex biomimetic trabecular bone-like structures possessing 3-D macropore architectures while minimizing material waste [20,21,22]. However, current clinical surface modification treatments that use line-of-sight-based processes, such as grit blasting, to roughen the surfaces of Ti alloys are incapable of accessing the internal surfaces of macroporous AM implants. New, non-line-of-sight approaches are needed that are capable of tailoring, down to the nanoscale, the roughness of internal Ti and Ti alloy surfaces within porous implants produced by AM to promote bone in-growth and improve integration.

In this paper, a new, non-line-of-sight, gas/solid-reaction-based approach [23] is demonstrated for generating thin (submicron), continuous, tailorable, nanoporous/nano-rough Ti-rich surfaces on smooth (polished) or microrough (grit-blasted, acid-etched) AM Ti alloy (TiAl6V4) substrates, with the resulting surfaces exhibiting enhanced in vitro osteoblastic responses from MSCs. This surface reaction process involves the use of:

(1) A modest thermal oxidation treatment in air to generate a thin (submicron), conformal, continuous TiO_2_-based surface layer of tailorable thickness on the TiAl6V4 alloy.
(2) Selective reaction with Ca vapor for reduction of the TiO_2_ in the thin surface layer to yield a conformal, thin, nanoporous Ti-based layer, along with CaO.
(3) Selective dissolution of the CaO product to yield a conformal, thin, nanoporous/nanorough Ti-based surface layer bonded to the underlying dense TiAl6V4 alloy.

The aims of this paper are: (i) to demonstrate a calciothermic reduction (CaR)-based process for generating consistent and reproducible nanoporosity/nanoroughness on TiAl6V4 alloy surfaces, (ii) to evaluate the mechanism of formation of such surface nanoporosity/nanoroughness, and (iii) to provide an initial in vitro evaluation of the osteoblastic responses of MSCs cultured on CaR-modified micro/nanorough surfaces.

## 2. Materials and Methods

### 2.1. Titanium Alloy Substrate Preparation

Titanium-aluminium-vanadium alloy disks (15 mm diameter, 4 mm thick) were generated by direct metal laser sintering (DMLS) of plasma-atomized Ti-Al(6 wt%)-V(4 wt%) powder (grade 5 TiAl6V4, Advanced Powders & Coatings, Quebec, Canada) [18]. After atomization, the TiAl6V4 particles were sorted by sieving to the 25–45 µm diameter range (as per the ISO 13485 certification for medical devices) [24]. DMLS was conducted with a continuous 200 W ytterbium-doped fibre (1054 nm) laser system (EOS, EmbH Munchen, Germany) using a laser scanning speed of 7 m s^−1^ with a step size of 100 μm and a laser spot size of 0.1 mm. The disks were removed from the build plate by electrical discharge machining. The average porosity of the disks was 4.2% based on the Archimedes test using water as the buoyant fluid.

Some of the TiAl6V4 disk surfaces were grit-blasted using calcium phosphate particles (AB Dental, Ashdod, Israel) and rinsed three times with ultra-pure water at room temperature. The disk surfaces were etched with ultrasonication in a 0.3 N nitric acid solution for 5 min at 45 °C, followed by rinsing two times (5 min each) in ultra-high purity water and then in 97% methanol for 5 min at 25 °C. A pickling treatment was conducted, which consisted of three 10 min ultrasonic rinses in ultrapure water, followed by immersion in an 1:1 aqueous solution of 20 g L^−1^ NaOH and 20 g L^−1^ H_2_O_2_ for 30 min at 80 °C, and ultrasonication in the ultrapure distilled H_2_O for 10 min [18]. The disk surfaces were cleaned in an industrial degreaser for 12 min, and then immersed in 65% aqueous nitric acid at 100 °C for 10 min, followed by rinsing three times in ultrapure distilled water for 10 min. After rinsing, the surfaces were blotted with lint-free laboratory wipes and dried in ambient air at 25 °C. These grit-blasted and etched disks are referred to herein as the “microrough” or “rough” TiAl6V4 specimens. Samples were wrapped in aluminium foil and packaged in plastic bags before sterilization by gamma irradiation.

Some of the DMLS-sintered TiAl6V4 disks were ground and polished using a series of silicon carbide abrasive papers (320 and 600 ANSI grit, Allied High Tech Products, Inc., Rancho Dominguez, CA, USA), diamond polycrystalline pastes (9 μm and 3 μm, Allied High Tech Products, Inc.), and colloidal silica (0.05 μm, Allied High Tech Products, Inc.). A METPREP 3 Grinder/Polisher with PH-3 (Allied High Tech Products, Inc.) was used for the grinding and polishing. A contra rotation mode with a sample rotation speed of 20 rpm and a single force mode were selected for all grinding and polishing steps. For the 320 grit SiC abrasive, a 150 rpm platen speed and 4 lbf of force were applied with water for 10 min. For the 600 grit SiC abrasive, a 120 rpm platen speed and 4 lbf of force were applied with water for 10 min. For the 9 μm diamond polycrystalline paste, a Plan-B pad (Allied High Tech Products, Inc.) was used, and a 100 rpm platen speed and 3 lbf of force were applied with GreenLube (Allied High Tech Products, Inc.) for 10 min. For the 3 μm diamond polycrystalline paste, a DiaMat pad (Allied High Tech Products, Inc.) was used, and a 100 rpm platen speed and 2 lbf of force were applied with GreenLube for 10 min. For the colloidal silica, a Chem-Pol pad (Allied High Tech Products, Inc.) was used, and 80 rpm platen speed and 2 lbf of force were applied with water for 10 min. The disks were then ultrasonically cleaned in acetone and ethanol for 10 min each at 25 °C. These polished disks are referred as the “smooth” or “polished” TiAl6V4 specimens.

### 2.2. Oxidation, Calciothermic Reaction, and Oxide Dissolution

**Figure 1:**
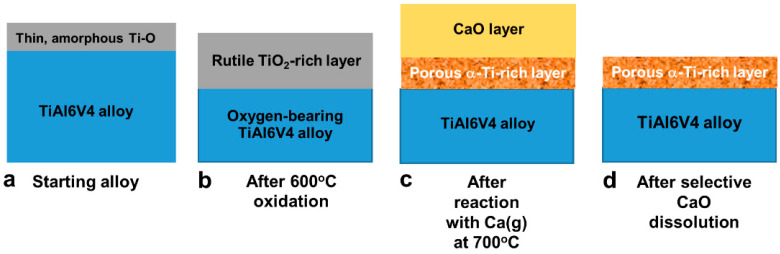
Schematic illustrations of the chemical, phase, and structural evolution associated with the non-line-of-sight reaction process: (**a**) starting TiAl6V4 alloy, (**b**) after surface oxidation in air at 600 °C for 6 h, (**c**) after reaction with Ca(g) at 700 °C for 1 h, (**d**) after selective CaO dissolution in acetic acid.

The generation of nanoporous/nanorough Ti-based surfaces on dense, smooth (polished) direct metal laser-sintered (DMLS) TiAl6V4 specimens, and on dense, microrough (grit-blasted, acid-etched) DMLS TiAl6V4 specimens, was accomplished via the following sequential non-line-of-sight steps [23] illustrated in Figure 1:

(i) Exposure to air at 600 °C for 6 h to generate a continuous, conformal, nanocrystalline rutile TiO_2_-based surface layer on the TiAl6V4 surface (Figure 1a,b).
(ii) Reaction of the TiO_2_-based layer with Ca(g) at 700 °C for 1 h to generate CaO and Ti-rich products (Figure 1c).
(iii) Exposure to a 3 M acetic acid solution for 40 min at 25 °C to selectively dissolve the CaO reaction product to yield a thin, porous, conformal Ti-rich surface layer (Figure 1d).

To accomplish these steps, TiAl6V4 disks were placed in a rectangular alumina crucible (107 mm × 30 mm × 15 mm, 99.8% purity, Almath Crucibles ltd, Newmarket, UK) and then heated at 5 °C min^−1^ in ambient air to 600 °C and held at this temperature for 6 h to form a thin surface oxide layer [25]. The oxide-layer-bearing TiAl6V4 disks were then allowed to undergo reaction with calcium vapor inside sealed metal ampoules. A given oxide-layer-bearing disk was placed in a small rectangular crucible (20 mm × 20 mm × 5 mm) made of titanium foil (>99.9% purity, 0.1 mm thick, MTI Corporation, Richmond, CA, USA). Titanium foil crucibles of similar dimensions containing calcium granules (1.0 g, 99.5% purity, <3.36 mm, Alfa Aesar, Tewksbury, MA, USA) were also prepared. Four titanium crucibles containing oxide-layer-bearing TiAl6V4 disks were placed, in an alternating manner, with five calcium-granule-bearing titanium crucibles inside a low-carbon steel tube (3.6 cm internal diameter × 29.2 cm long x 1.2 mm thick, 1008/1010 steel, McMaster-Carr, Elmhurst, IL, USA) (Supplemental Figure S1). Both ends of the steel tube were crimped shut and then sealed by welding within an argon atmosphere (<3.0 ppm O_2_, <3.0 ppm H_2_O) glove box. The specimen-bearing steel ampoule was then heated in a flowing (154 mL min^−1^) high-purity argon (99.999% purity, 1 ppm O_2_, Airgas, Radnor, PA, USA) atmosphere at 5 °C min^−1^ to 700 °C and held at this temperature for 1 h, followed by cooling at 5 °C min^−1^ to 25 °C. During such heating, calcium vapor generated inside the sealed steel ampoule underwent selective reaction with the thin oxide layer present on each TiAl6V4 specimen. After removal from the steel ampoule, the disks were immersed in 40 mL of a 3 M acetic acid solution for 40 min at 25 °C to selectively dissolve the CaO from the reacted specimens [26]. The disks were then ultrasonically cleaned for 10 min each in acetone, ethanol, and de-ionized water (resistivity = 18.2 MΩ at 25 °C). The samples were then dried under vacuum (−100 kPa) for 15 min at 25 °C. The resulting samples were stored in the argon glove box mentioned above.

### 2.3. Surface Characterization

Images of specimen surfaces at various stages of reaction were obtained using scanning electron microscopy (secondary electron, SE, images: Nova FE-SEM, FEI Co., Hillsboro, OR, USA; backscattered electron, BSE, images: Quanta FE-SEM, FEI Co.). SE images were acquired using an acceleration voltage and current of 5 kV and 0.11 nA, respectively. BSE images and energy dispersive X-ray (EDX) analyses for elemental mapping were acquired using an Oxford INCA Xstream-2 silicon drift detector with a Xmax80 window with an acceleration voltage and current of 10 kV and 16–48 nA, respectively. Specimen cross-sections were prepared by focused ion beam milling (Quanta 3D FEG, FEI Co., Hillsboro, OR, USA) at 30 kV and 30 pA after deposition of a top Pt layer at 20 kV and 0.3 nA. Specimen cross-sections were transferred to a copper grid to allow for transmission electron microscopy (Talos 200X, FEI Co.). Elemental maps of such cross-sections were acquired under STEM mode with EDX analyses. X-ray diffraction (XRD, D2 PHASER, Bruker, Madison, WI, USA) results were acquired using Cu Kα radiation (1.54184 Å) with 0.05° increments and a 0.2 s dwell time per step.

For the smooth (polished) specimens, atomic force microscopy [27] (NanoScope IIIa, Digital Instrument, Veeco Metrology Group, Santa Barbara, CA, USA) was conducted using a n-doped Si cantilever with a resonance frequency of 300 kHz (Bruker, Billerica, MA, USA). A scan rate of 0.493 Hz was used with 512 lines. Five analyses, each conducted over 65 μm × 65 μm, were used to obtain average values and 95% confidence intervals of surface roughness (Ra) and peak-to-valley height.

The microscale surface roughness was assessed by laser confocal microscopy (LCM, Zeiss LSM 710, Carl Zeiss Microscopy GmbH, JenaGmhHJena, Germany) [28]. Z-stacks were obtained with a Plan Apochromat 20×/0.8 M27 objective with a 1x optical zoom, using a 405 nm laser in reflection mode at 50% power. Scanning was conducted with a 0.39 μs pixel dwell, a 25 μm pinhole, a 420.9 μm × 420.9 μm image size, and a step size of 1 μm. The mean and standard errors were obtained for 12 samples per measurement and 2 surfaces per specimen type and presented as mean and 95% confidence intervals. 

Contact angle values were obtained by goniometer measurements (CAM 250, Rame-Hart) using 2 µL of water at each of 6 different locations, with drying conducted using nitrogen between each measurement. After applying an ultrapure distilled water droplet, the contact angle was measured every 5 s for 20 s. The resulting 4 measurements at that location were then averaged to obtain 1 of the 6 measurements per disc for 2 discs.

### 2.4. Cell Culture

Human female bone marrow stromal cells (MSCs) (Donor #8011L, Texas A&M Institute for Regenerative Medicine, College Station, TX, USA) were cultured in MSC growth medium (GM), comprised of αMEM with 4 µM L-glutamine and 16.5% foetal bovine serum, at 37 °C in 5% CO_2_ and 100% humidity [29]. The MSCs were cultured to confluence in T75 flasks (Corning Inc., Oneonta, NY, USA) before plating on a surface. For biological analysis, surfaces were placed face down in the biological safety cabinet and sterilized by UV light for 24 h. The surfaces were then turned face up and again sterilized for another 24 h. The surfaces were then placed face up in 24-well plates, and cells were plated at a density of 20,000 cells mL^−1^ at 0.5 mL per well. MSCs cultured on tissue culture polystyrene (TCPS) served as experimental controls. The GM was changed 24 h after plating, with media changes conducted every subsequent 48 h for up to 7 days. At day 7, cells were incubated for 24 h with fresh GM before being harvested. (Note: Prior work has demonstrated that bone marrow stromal cells differentiate after 7 days, without the addition of factors or hormones, if the cells are seeded on microroughened surfaces [29].) Upon harvesting, conditioned media were subsequently collected and stored at −80 °C. MSCs were rinsed twice with 1x PBS, placed in 0.5 mL of Triton-X100, and stored at −80 °C for biological assays.

### 2.5. Analyses of Cellular Responses

Cell layers were lysed by ultrasonication at 40 V for 15 s well^−1^ (VCX 130; Vibra-Cell, Newtown, CT, USA). The QuantiFluor* dsDNA system (Promega, Madison, WI, USA) was used to determine total DNA content by fluorescence. Enzyme-linked immunosorbent assays were used to determine the levels of osteogenic factors in the conditioned media. OPN, BMP2, BMP4, OPG, VEGF (R&D Systems, Inc., Minneapolis, MN, USA) and OCN (ThermoFisher Scientific, MN, USA) were quantified according to the manufacturer’s protocol.

The relative influences of micro-to-nanoscale roughness on osteoblastic responses of the MSCs were examined by evaluating the responses on 4 types of titanium alloy surfaces:

(i) TiAl6V4 surfaces polished to a 0.05 μm finish (“polished“ specimens)
(ii) TiAl6V4 surfaces exposed to grit blasting and acid etching (“rough“ specimens)
(iii) Polished TiAl6V4 surfaces that had undergone the calciothermic reaction-based process (“P-CaR“ specimens).
(iv) Grit-blasted/acid-etched (rough) TiAl6V4 surfaces that had undergone the reaction process (“R-CaR“ specimens)

### 2.6. Statisical Analyses

Data are provided as means ± standard error of the mean of six independent cultures/variable. All experiments were repeated to ensure the validity of observations, with results from individual experiments shown. Statistical analysis within a group was performed by one-way analysis of variance (ANOVA), and multiple comparisons between the groups were conducted with a two-tailed Tukey correction [30]. A *p* value ≤ 0.05 was considered statistically significant. Such statistical analyses were performed with GraphPad Prism version 5.04.

## 3. Results

### 3.1. Nanochemical Modification of the TiAl6V4 Surface

**Figure 2:**
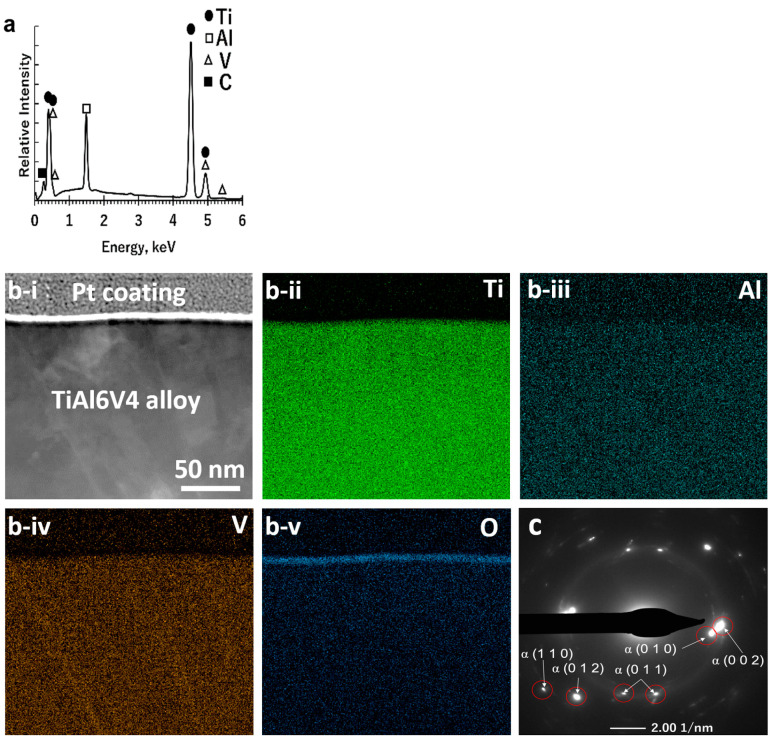
Characterization of a starting polished DMLS TiAl6V4 specimen: (**a**) SEM/EDX spectrum obtained from top-down analysis of the external surface; (**b-i**) cross-sectional STEM image and associated EDX elemental maps for (**b-ii**) titanium, (**b-iii**) aluminium, (**b-iv**) vanadium, and (**b-v**) oxygen obtained from an ion-milled cross-section at the surface, revealing a thin Ti-O-rich surface layer; (**c**) SAED pattern, consistent with the α-TiAl6V4 phase, obtained from an ion-milled cross-section.

Elemental maps obtained from SEM/EDX analyses of the polished surface of the starting TiAl6V4 alloy indicated a uniform distribution of titanium (Ti), aluminium (Al), and vanadium (V) (Figure 2a, Supplemental Figure S2 and Table S1). STEM/EDX analyses (Figure 2b) of an ion-milled cross-section at the alloy surface revealed the presence of a thin (6 ± 2 nm) native Ti-O-rich surface layer (as illustrated in Figure 1a). XRD analysis of the polished surface (Supplemental Figure S3) of the starting alloy, along with SAED analysis (Figure 2c) and high-resolution electron microscopy (HRTEM, Supplemental Figure S4a) of an ion-milled cross-section of the starting alloy, indicated the predominant presence of the α (hexagonal) polymorph of the TiAl6V4 alloy.

**Figure 3:**
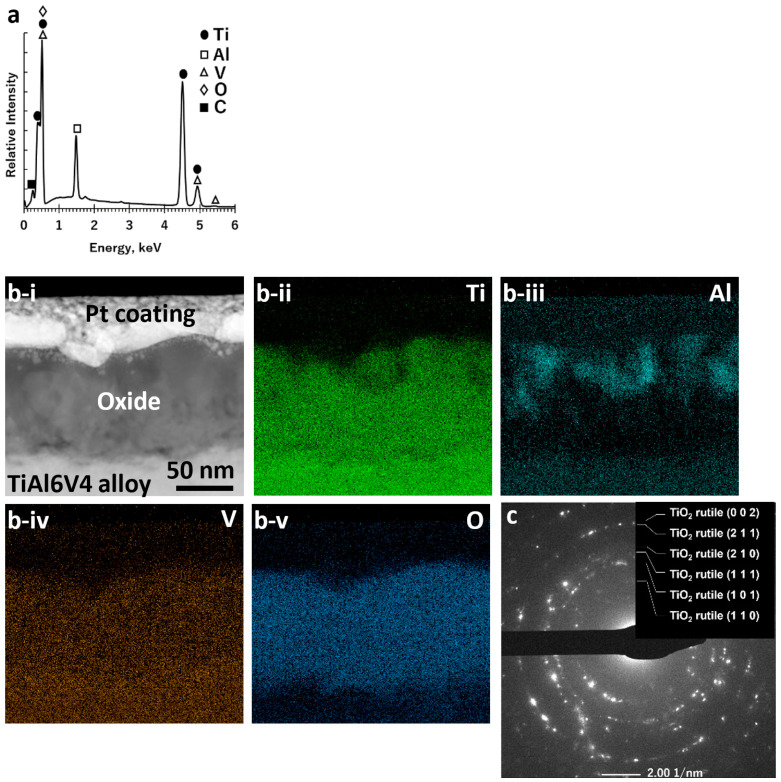
Characterization of a polished DMLS TiAl6V4 specimen after exposure to air at 600 °C for 6 h: (**a**) SEM/EDX spectrum obtained from top-down analysis of the external surface; (**b-i**) cross-sectional STEM image and associated EDX elemental maps for (**b-ii**) titanium, (**b-iii**) aluminium, (**b-iv**) vanadium, and (**b-v**) oxygen, obtained from an ion-milled cross-section at the surface, revealing the Ti-O-rich surface layer; (**c**) SAED pattern, consistent with rutile TiO_2_, obtained from an ion-milled cross-section at the surface.

After exposure to air for 6 h at 600 °C, the alloy surface was covered with a continuous, conformal, nanocrystalline rutile TiO_2_-based layer (Figure 1b), as confirmed by SEM/EDX analyses (Figure 3a; Supplemental Figure S5, Supplemental Table S1) of the specimen surface and by scanning transmission electron microscopy (STEM) with EDX analyses, SAED, and high-resolution transmission electron microscopy (HRTEM) of ion-milled cross-sections (Figure 3b,c, Supplemental Figure S4b). The average thickness of this oxide layer was 90 ± 16 nm. The average specimen mass gain per area, Δm/A, resulting from this oxidation treatment was 5.7 ± 0.2 × 10^−5^ g cm^−2^. After surface oxidation, additional XRD peaks appeared adjacent to (at lower 2θ values than) diffraction peaks for the α-TiAl6V4 phase (Supplemental Figure S3b).

**Figure 4:**
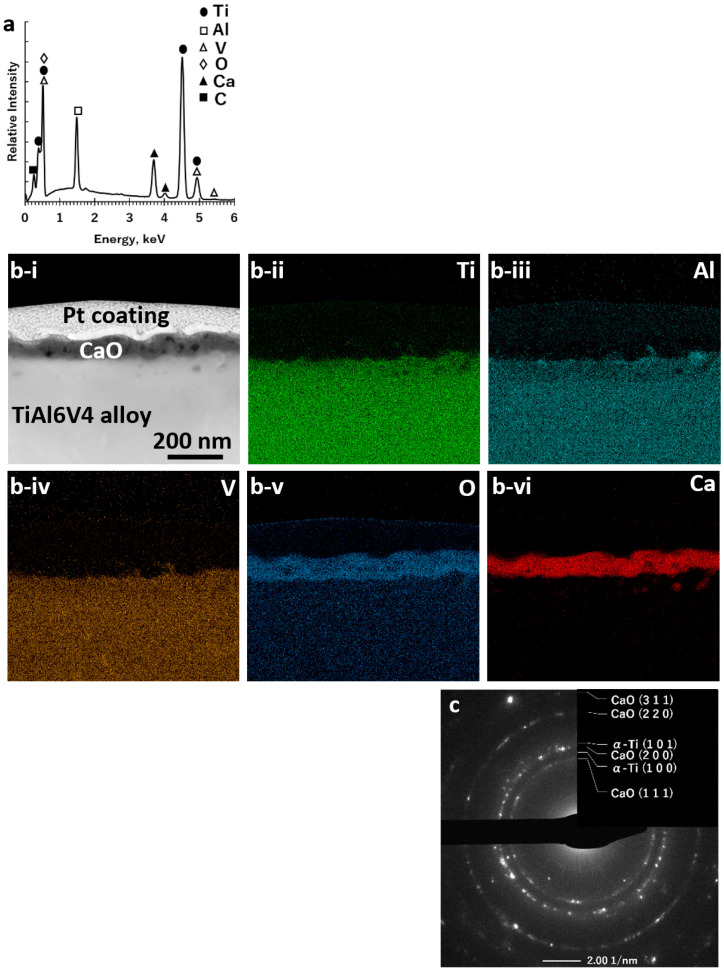
Characterization of a polished, oxidized DMLS TiAl6V4 specimen after calciothermic reaction at 700 °C for 1 h: (**a)** SEM/EDX spectrum obtained from top-down analysis of the external surface; (**b-i**) cross-sectional STEM image and associated EDX elemental maps for (**b-ii**) titanium, (**b-iii**) aluminium, (**b-iv**) vanadium, (**b-v**) oxygen, and (**b-vi**) calcium obtained from an ion-milled cross-section at the surface; (**c**) SAED pattern, consistent with the presence of CaO and an α-Ti-rich phase, obtained from an ion-milled cross-section at the surface.

After reaction with Ca(g) for 1 h at 700 °C, the TiO_2_ surface layer was converted into nanocrystalline CaO and Ti products (Figure 1c), as confirmed by SEM/EDX analysis of the specimen surface (Figure 4a; Supplemental Figure S6, Supplemental Table S1), and by SAED and HRTEM analyses of ion-milled cross-sections (Figure 4b,c, Supplemental Figure S4c,d). STEM/EDX analyses of ion-milled cross-sections (Figure 4b) revealed an external CaO-rich product layer with an average thickness of 77 ± 16 nm residing above an underlying Ti-rich layer. The average specimen mass gain per area resulting from this calciothermic reaction treatment was 1.7 ± 0.2 × 10^−5^ g cm^−2^.

**Figure 5:**
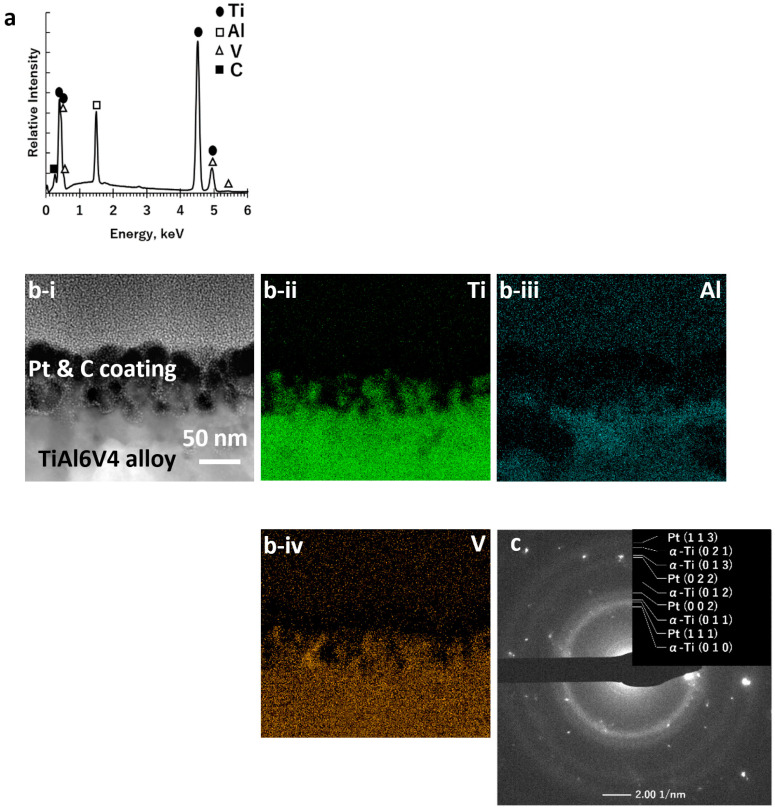
Characterization of the polished, oxidized, and calciothermically reacted DMLS TiAl6V4 specimen after selective CaO dissolution: (**a**) SEM/EDX spectrum obtained from top-down analysis of the external surface; (**b-i**) cross-sectional STEM image and associated EDX elemental maps for (**b-ii**) titanium, (**b-iii**) aluminium, and (**b-iv**) vanadium obtained from an ion-milled cross-section at the surface; (**c)** SAED pattern, consistent with an α-Ti-rich phase, obtained from an ion-milled cross-section at the surface.

Selective dissolution of the CaO product phase in acetic acid yielded a porous Ti-rich, surface layer (Figure 1d), as confirmed by SEM/EDX analyses (Figure 5a; Supplemental Figure S7, Supplemental Table S1). STEM/EDX, SAED, and HRTEM analyses of ion-beam milled cross-sections at the surface of this specimen (Figure 5b,c, Supplemental Figure S4e) revealed a nanoporous (CaO-free) surface layer with an average thickness of 51 ± 20 nm comprised of a nanocrystalline α-Ti-rich phase.

Similar changes in surface chemistry and phase content were observed after each stage of the reaction process with microrough (grit-blasted, acid-etched) DMLS TiAl6V4 specimens, as indicated by SEM/EDX and XRD analyses in Supplemental Figures S8–S12.

### 3.2. Nanostructural Modification of the TiAl6V4 Surface

**Figure 6:**
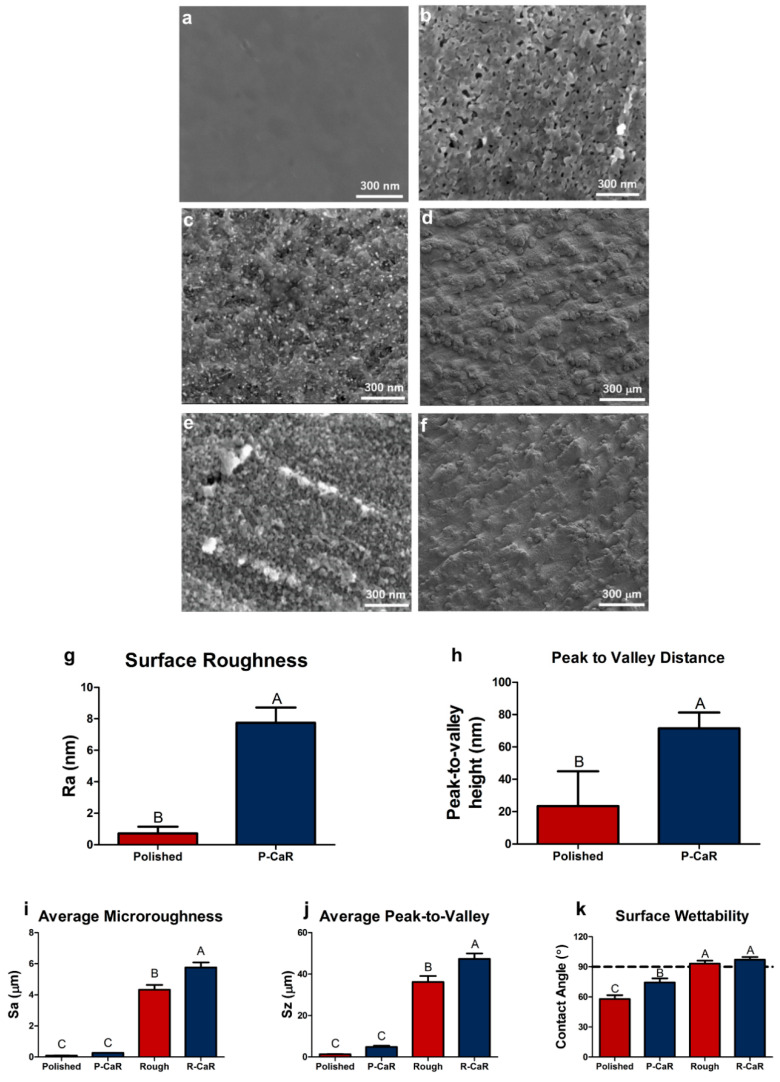
Characterization of the surface topography of polished and microrough (grit-blasted, acid-etched) DMLS TiAl6V4 specimens before and after the reaction process: (**a**) SE image of a starting polished specimen, (**b**) SE image of a polished specimen after completion of the surface reaction process (surface oxidation, reaction with Ca(g), CaO dissolution), (**c**,**d**) SE images of a starting microrough specimen, (**e**,**f**) SE images of a microrough specimen after completion of the reaction process, (**g**,**h**) AFM analysis of the average nanoscale roughness (Ra) and peak-to-valley height for polished specimens before and after completion of the surface reaction process, (**i**) LCM analyses of the average microscale roughness (Sa) for polished and microrough specimens before and after completion of the surface reaction process, (**j**) LCM analyses of the average microscale peak-to-valley distance (Sz) for polished and microrough specimens before and after completion of the surface reaction process, (**k**) water contact angle analyses (sessile drop measurements) of polished and microrough specimens before and after completion of the surface reaction process. Differences in these data were deemed to be statistically significant (with such significant differences indicated by different letter labels) for *p* values ≤ 0.05.

After exposure to the reaction process (surface oxidation, calciothermic reaction, calcia dissolution), the smooth surfaces of polished DMLS TiAl6V4 specimens were converted into nanoporous/nanorough surfaces (Figure 1d) as confirmed by SEM (Figure 6a,b) and AFM analyses (Figure 6g,h). AFM analyses indicated that the reaction process resulted in a statistically significant increase in average surface roughness. For microrough TiAl6V4 specimens, the change in nanoscale surface roughness (Figure 6c,e) did not result in a reduction in microscale roughness (Figure 6d,f). Indeed, LCM analyses (Figure 6i,j) indicated that the reaction process resulted in small changes in microscale roughness (from 4 ± 0.4 μm to 6 ± 0.4 μm) and peak-to-valley height (from 36 ± 3 μm to 47 ± 3 μm). Whereas exposure of polished TiAl6V4 surfaces to this reaction process resulted in an increase in water contact angle (Figure 6k), microrough and reaction-modified microrough TiAl6V4 surfaces exhibited similar contact angles, although these latter values were higher than for polished and reaction-modified polished specimens.

### 3.3. Responses of MSCs to Reaction-Modified TiAl6V4 Surfaces

**Figure 7:**
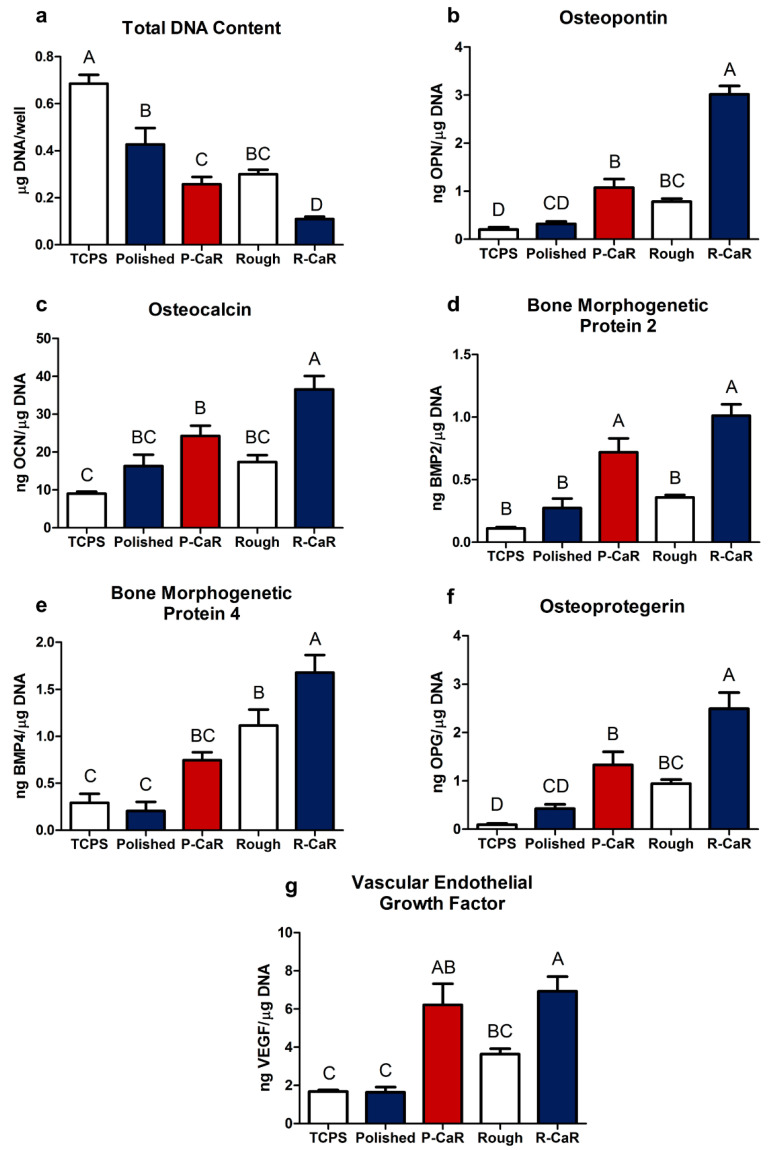
Responses of MSCs to reaction-modified TiAl6V4 surfaces: (**a**) total DNA content, (**b**) osteopontin content, (**c**) osteocalcin content, (**d**) BMP 2 content, (**e**) BMP 4 content, (**f**) OPG content; (**g**) VEGF-A content. “TCPS” refers to tissue culture polystyrene. “Polished” refers to a polishedTiAl6V4 surface. “Rough” refers to a TiAl6V4 surface that had undergone grit blasting and acid etching. “P-CaR” refers to a polished TiAl6V4 specimen that had undergone the surface reaction process. “R-CaR” refers to a grit-blasted/acid-etched TiAl6V4 surface that had undergone the surface reaction process. Data are shown as the mean ± standard error mean, for an N = 6 per group, and represented 3 experimental repeats. Differences in these data were deemed to be statistically significant (with such significant differences indicated by different letter labels) for *p* values < 0.05.

Cells on different surfaces possessed similar morphologies, although reduced levels of confluence were observed on reaction-modified microrough TiAl6V4 surfaces. MSCs cultured on reaction-modified polished or reaction-modified microrough TiAl6V4 surfaces exhibited enhanced expression of the osteoblast phenotype relative to non-modified TiAl6V4 surfaces. The values of total DNA content from cells cultured on all TiAl6V4 surfaces were lower than for TCPS, as reported in prior work [31], although no significant difference in total DNA content was observed between cells cultured on the starting polished or starting microrough TiAl6V4 surfaces. However, the total DNA content from cells cultured on reaction-modified surfaces was reduced compared to cells on non-modified surfaces (Figure 7a). Osteopontin and osteocalcin levels were higher from cells cultured on reaction-modified microrough surfaces than for non-modified microrough surfaces, with an increase in the osteopontin content also observed from cells on reaction-modified polished surfaces relative to non-modified polished surfaces (Figure 7b,c). BMP2, OPG, and VEGF contents were all higher from cells on reaction-modified polished surfaces than on non-modified polished surfaces, and from cells on reaction-modified microrough surfaces than on non-modified microrough surfaces (Figure 7d–g). The BMP4 content of the conditioned media was also higher for cells on reaction-modified microrough substrates relative to non-modified microrough substrates (Figure 7e).

It is worth noting that the total DNA, osteopontin, osteocalcin, BMP4, OPG, and VEGF levels from MSCs cultured on reaction-modified polished TiAl6V4 surfaces were similar to assay results for non-modified microrough surfaces (Figure 7e–g). The BMP2 content from cells cultured on reaction-modified polished surfaces was also higher than for non-reacted microrough surfaces.

## 4. Discussion

### 4.1. Thermodynamic Considerations

The thin continuous rutile TiO_2_-based surface layers that had formed on the polished and microrough DMLS TiAl6V4 surfaces after the 600 °C oxidation treatment were converted into a mixture of CaO and Ti-rich products via the following net displacement reaction with Ca at 700 °C:

$$2\mathrm{Ca}(g)+\mathrm{Ti}O_{2}(s)\to 2\mathrm{CaO}(s)+\mathrm{Ti}(s)$$

Calcium and titanium are chemically compatible at 700 °C; that is, no stable Ca-Ti-bearing compounds have been reported at 700 °C [32]. From available thermodynamic data [33,34], and assuming that rutile TiO_2_(s), CaO(s), and Ti(s) are present in their pure component reference states and that Ca(g) behaves as an ideal gas (with a pure gas, 1 atm reference state), the equilibrium vapor pressure of Ca(g) associated with the above reaction at 700 °C was calculated to be 7.5 × 10^−11^ atm. The calculated vapor pressure of Ca(g) in equilibrium with pure solid Ca(s) at 700 °C was 1.5 × 10^−4^ atm [34]; that is, the equilibrium Ca vapor pressure generated from solid Ca at 700 °C was much larger than the value required for the net displacement reaction to proceed to the right. Hence, Ca vapor was chosen in this work as a highly effective and selective agent for converting thin rutile TiO_2_-based layers into CaO and Ti at a modest reaction temperature of 700 °C.

### 4.2. Evolution of Surface Chemistry and Phase Content

Electron microscopy coupled with EDX, XRD, SAED, and mass change analyses after each stage of the reaction process (surface oxidation, reaction with calcium vapor, calcia dissolution) were consistent with the changes in TiAl6V4 surface chemistry and phase content illustrated in Figure 1.

The starting TiAl6V4 alloy was comprised predominantly of the α (hexagonal) phase and contained a thin (6 nm) native Ti-O surface layer. The diffraction peaks obtained from the α-TiAl6V4 phase were shifted to higher 2θ values than the diffraction peaks for pure α-Ti (Supplemental Figure S3b) due to the lattice contraction that occurs upon alloying of titanium with aluminium and vanadium [35,36].

After oxidation in air for 6 h at 600 °C, a thicker (90 nm) continuous rutile titania-rich layer formed on the α-TiAl6V4 surfaces. Although Al-O-rich regions were observed near the outer surface of the rutile-based layer, crystalline Al_2_O_3_ was not detected by SAED analysis, which was consistent with the presence of some amorphous aluminium oxide [37]. Although readily detected by the STEM/EDX and SAED analyses of ion-milled cross-sections, the rutile titania-rich surface layer was too thin to allow for detection by top-down XRD analyses. However, given the penetration of Cu Kα X-rays through TiAl6V4, such XRD analysis was used to evaluate changes in the alloy below the thin titania-rich surface layer. (Note: As an example, the calculated penetration depth of 70% of incident Cu Kα X-rays, I/I_o_ = 0.70, into TiAl6V4 was over 4000 nm [38,39].) After the 600 °C oxidation treatment, additional XRD peaks adjacent to (at lower 2θ values than) those for the α-TiAl6V4 phase were detected, as has been reported to occur upon the incorporation of some dissolved oxygen into this alloy [40]. This oxygen incorporation in the alloy was also consistent with the measured mass gain per area (5.7 × 10^−5^ g cm^−2^) after the oxidation treatment. If this mass gain resulted solely from the oxygen present within the observed dense rutile TiO_2_-rich surface layer, then the associated thickness of such a layer (using a molar volume of 18.8 cm^3^ mol^−1^ for rutile TiO_2,_ PDF#: 00-021-1276 [39]) would be 335 nm. This calculated value was appreciably greater than the observed average layer thickness of 90 nm, which indicated that the oxidation treatment resulted in some oxygen incorporation into the underlying TiAl6V4 alloy, as well as in the formation of the rutile TiO_2_-rich surface layer.

After reaction with Ca(g) at 700 °C for 1 h, the rutile TiO_2_-rich surface layer was completely converted into an external CaO layer on top of an underlying α-Ti-rich layer. Whereas reaction temperatures of 650 °C and 750 °C were also examined, the lowest temperature at which the TiO_2_ layer was completely reduced by Ca(g) into CaO and Ti products within 1 h was found to be 700 °C. (Note: Longer times than 1 h at 700 °C were not examined for this calciothermic reaction step to minimize sintering and coarsening of the nanocrystalline α-Ti grains in the α-Ti layer.) Calciothermic reaction for 1 h at 700 °C resulted in an average mass gain of 1.7 × 10^−5^ g cm^−2^, which was attributed to the incorporation of Ca, in the form of CaO, onto specimen surfaces. This mass gain corresponded to the addition of 4.2 × 10^−7^ mol cm^−2^ of CaO. The thickness of a dense CaO layer associated with this mass gain was calculated to be 71 nm (using the CaO molar volume of 16.8 cm^3^ mol^−1^, PDF#: 01-070-5490 (39)), which was not far from the average measured CaO layer thickness of 77 ± 16 nm. Comparison of the XRD patterns obtained before and after this calciothermic reaction step indicated the loss of some diffraction peaks (e.g., the peak located at a lower 2θ value relative to the (101) peak for α-TiAl6V4 was lost) consistent with the removal of some oxygen from the TiAl6V4 alloy during the CaO formation process; that is, some of the CaO product formed by the reaction of Ca(g) with dissolved oxygen removed from the TiAl6V4 alloy.

The CaO-bearing specimens were then exposed to acetic acid to allow for the selective dissolution of the CaO product to expose the underlying porous α-Ti-rich surface layer. The magnitude of the measured mass loss per area after this acid treatment was similar to the magnitude of the mass gain per area after the entire calciothermic reaction process, which indicated that the acetic acid treatment was effective in removing CaO via selective dissolution.

### 4.3. Mechanism of Nanopore Formation on the TiAl6V4 Surface

Global mass change and local thickness measurements were used to evaluate the mechanism of nanopore formation on the α-TiAl6V4 surfaces. As discussed above, the reaction of the polished and surface oxidized (TiO_2_-bearing) TiAl6V4 specimens with Ca(g) resulted in an average mass gain which corresponded to the addition of 4.2 × 10^−7^ mol cm^−2^ of CaO to the specimen surfaces. For every 2 moles of CaO formed by the reaction, 1 mole of Ti should be generated from TiO_2_. The average thickness of a dense Ti layer corresponding to this CaO addition was calculated to be 20 nm (using the α-Ti molar volume of 10.6 cm^3^ mol^−1^, PDF#: 00-044-1294 [39]). However, the average measured thickness of the α-Ti-rich layer was 51 ± 20 nm, which was consistent with a relative porosity for this layer of 54 ± 18% (=100 × {1 − [20/(51 ± 20)]}). This simple estimate of the apparent porosity value of the α-Ti-rich surface layer was not far from the calculated loss in solid volume upon reducing rutile TiO_2_ into α-Ti (44%); that is, this relative porosity value was consistent with the conversion of a dense, conformal TiO_2_-based surface layer (via oxygen removal) into a nanoporous, conformal α-Ti-based surface layer under the external layer of CaO. An important consequence of this nanopore formation mechanism is that the thickness of the conformal nanoporous/nanorough Ti-based layer generated on TiAl6V4 surfaces is directly proportional to the thickness of the conformal TiO_2_ layer formed on such surfaces which, in turn, can be readily tailored at the submicron level by adjusting the initial modest conditions for surface oxidation.

### 4.4. Interactions of MSCs with Reaction-Modified Surfaces

MSCs are highly sensitive to implant surface characteristics [41,42]. In vitro studies using clinically available implant surfaces have shown that cells cultured on both Ti and Ti alloy implants possessing structural features at the micro-scale and meso-scale exhibit enhanced osteogenic behaviour (e.g., increases in BMP2, BMP4, and osteoprotegerin levels, in the absence of osteogenic induction media), which, in turn, correlates strongly with overall implant success [43,44]. In the present work, MSCs cultured on the reaction-modified microrough TiAl6V4 surfaces exhibited a significantly enhanced production of osteopontin and osteocalcin, which are two potent markers of osteoblast differentiation and maturation [44,45], relative to MSCs cultured on non-modified microrough surfaces. The osteopontin production of MSCs on polished surfaces also increased after surfaces were exposed to the reaction process.

During osseointegration, bone marrow stromal cells differentiate to became osteoblasts which, in turn, leads to bone formation and integration of the implant. It has been well characterized that as cells differentiate, their rate of proliferation decreases. In the present study, as expected, the MSCs grown on reaction-modified surfaces had decreased cell proliferation as measured by DNA content. At the same time, the MSCs exhibited increased protein expression associated with osteogenesis, indicating that these cells were undergoing differentiation to become osteoblasts [31,46].

Osteogenesis is not only dependent on stem cell differentiation, but also the paracrine signalling factors produced by these cells that regulate cells away from the implant. The osteogenic signalling proteins, BMP2, OPG, and VEGF-A, produced from cells on the reaction-modified microrough and polished surfaces were all higher than from cells on non-modified surfaces, with enhanced BMP4 production also observed for cells cultured on reaction-modified microrough surfaces. Elevated levels of soluble BMP2 and BMP4 have been correlated to increased osteogenesis [47,48]. BMP2 has been used previously to differentiate MSCs into osteoblasts in vitro, and BMP variants are currently used clinically to augment bone formation during spinal fusions and craniofacial bone augmentations [49,50,51]. Surfaces that have increased the production of potent bone induction proteins, such as BMP2 and BMP4, have high long-term implant retention rates over 10 years, with successful retentions occurring in 95.2% of cases [48]

Although nanoscale surface modifications can and do modulate cell fate, there has been considerable variation in their effectiveness for supporting osteogenesis in vitro and in vivo [52,53,54,55]. The majority of studies have been conducted using osteogenic media, which contains supplements that increase the calcium phosphate ion product in the media, leading to dystrophic calcification [56,57]. Thus, from these studies, it is difficult to identify the specific effects of individual nanoscale structures on the mechanisms driving the differentiation.

Surface wettability can also be an important factor for cellular attachment and differentiation [58,59]. The reaction-modified polished TiAl6V4 surfaces exhibited a higher water contact angle than the starting polished TiAl6V4 surfaces, even though the nanoscale surface roughness also increased. This result was inconsistent with the Wenzel equation [60,61], which predicts that the apparent contact angle of a liquid on a roughened solid surface, θ*, should decrease as the surface roughness increases (for cases where the equilibrium contact angle, θ, of the liquid on the flat solid is less than 90°). However, use of the Wenzel equation assumes that the liquid completely penetrates all cavities on the roughened surface. As has been reported for several biological systems (e.g., lotus leaves, legs of the water strider [62,63,64]), a non-wetting (θ* > 90°) metastable Cassie state can occur on certain nanostructured surfaces even if θ < 90°. Such a Cassie state, associated with small pockets of gas trapped within fine cavities below the liquid, can occur with fine surface cavities exhibiting re-entrant curvature (i.e., an increase in cavity diameter with increasing depth from the external surface) [65,66,67,68]. Whereas some of the surface nanopores seen in the cross-section in the STEM image in Figure 5b–i appear to exhibit such re-entrant curvature, further work (beyond the scope of this paper) would be needed for statistical evaluation of re-entrant curvature associated with such surface nanopores. Nonetheless, cells cultured on the reaction-modified polished TiAl6V4 surfaces of lower water contact angle exhibited similar or enhanced osteogenic responses relative to cells cultured on the non-modified polished surfaces. Cells cultured on the reaction-modified microrough surfaces also exhibited significantly enhanced osteogenic responses relative to non-modified microrough surfaces.

The novel reaction-based surface treatment of the present work [23] synergistically enhanced the osteogenic potential of microroughened TiAl6V4 implant surfaces. The non-line-of-sight nature of this process can also allow enhanced osteogenic behaviour on interior surfaces of 3-D macroporous AM implants, with apparent independence of interior surface microstructure, as indicated by elevated osteogenic soluble protein production on both polished and microroughened surfaces that were treated by this approach. Moreover, the present results demonstrate that this surface treatment is capable of sustaining cell growth and is not cytotoxic, as indicated by the robust increase in osteogenic factors and similar reduction in DNA content over 7 days relative to the published literature [31,46]. In light of these in vitro analyses, further work is underway to validate these results in preclinical and clinical studies.

## 5. Conclusions

A new non-line-of-sight, gas/solid reaction-based process was used to generate thin, continuous, conformal, nanorough α-Ti-based surface layers on polished or micro-rough surfaces of dense, direct-metal-laser-sintered TiAl6V4 specimens for use in orthopaedic and dental implant applications. A thin (90 nm) dense rutile TiO_2_-based surface layer formed by modest thermal oxidation (600 °C, 6 h) of a TiAl6V4 alloy underwent a reaction with heated Ca(g) (700 °C, 1 h) to yield an external CaO scale on an α-Ti-based layer on the TiAl6V4 alloy. Selective CaO dissolution then yielded a thin (51 nm) nanoporous α-Ti-based surface layer on the TiAl6V4 alloy. The conformal nature of the thin nanoporous α-Ti-based layer enabled separate control of roughness at the nanoscale and microscale; that is, nanoscale roughness could be superimposed on a previously microroughened TiAl6V4 surface without the loss of such microscale roughness.

MSCs cultured on the nanoroughened TiAl6V4 surfaces exhibited a significantly enhanced production of osteopontin (a potent marker of osteoblast differentiation and maturation) relative to MSCs cultured on surfaces that had not been exposed to the new gas/solid reaction process. The production of paracrine osteogenic signalling proteins (BMP2, osteoprotegerin, VEGF-A) by MSCs also increased on the reaction-modified surfaces. Even smooth (polished) TiAl6V4 surfaces exposed to this process yielded osteoblastic MSC responses similar or enhanced relative to MSCs cultured on TiAl6V4 surfaces generated by only a standard line-of-sight-based process (grit blasting then acid-etching). However, unlike line-of-sight-based surface treatments, the gas/solid reaction process introduced here enables tailoring of internal implant surfaces, such as those present inside porous, additively manufactured TiAl6V4 implants.

## 6. Patent

The calciothermic reaction process reported herein for generating micro/nano-structured surfaces on biomedical devices was conceived by one of the corresponding authors of this paper (K.H.S.) and is the subject of a filed patent application [23].

## Data Availability

The data that support the findings of this study are available from the corresponding authors upon reasonable request.

## Supplementary Materials

Supporting information can be downloaded at: https://www.mdpi.com/article/10.3390/biomimetics7030117/s1. Figure S1: Schematic illustration of the alternating arrangement of oxidized TiAl6V4 specimens (in Ti crucibles) with Ca granules (also in Ti crucibles), all of which were sealed within a steel ampoule, for the calciothermic reaction step; Figure S2: Characterization of a starting polished DMLS TiAl6V4 specimen; Figure S3: XRD analyses of polished DMLS TiAl6V4 specimen surfaces after each reaction stage; Figure S4: TEM analyses at various stages of reaction of polished DMLS TiAl6V4 specimens; Figure S5: Characterization of a polished DMLS TiAl6V4 specimen after exposure to air at 600 °C for 6 h; Figure S6: Characterization of a polished, oxidized DMLS TiAl6V4 specimen after calciothermic reaction at 700 °C for 1 h;Figure S7: Characterization of a polished, oxidized, and calciothermically-reacted DMLS TiAl6V4 specimen after selective CaO dissolution; Figure S8: Characterization of a starting microrough (grit-blasted and acid-etched) DMLS TiAl6V4 specimen; Figure S9: Characterization of a microrough (grit-blasted and acid-etched) DMLS TiAl6V4 specimen after oxidation; Figure S10: Characterization of a microrough (grit-blasted and acid-etched), oxidized DMLS TiAl6V4 specimen after calciothermic reaction at 700 °C for 1 h; Figure S11: Characterization of a microrough (grit-blasted and acid-etched), oxidized, calciothermically-reacted DMLS TiAl6V4 specimen after selective CaO dissolution; Figure S12: XRD analyses of a starting microrough (grit-blasted, acid-etched) DMLS TiAl6V4 specimen; Table S1: Normalized compositions obtained from top-down SEM/EDX analyses of the surfaces of TiAl6V4 specimens at various stages of surface modification.
