# Supplementary material for: Tailoring of TiAl6V4 Surface Nanostructure for Enhanced In Vitro Osteoblast Response via Gas/Solid (Non-Line-of-Sight) Oxidation/Reduction Reactions
Source: Biomimetics (Basel). 2022 Aug 25;7(3):117. doi: 10.3390/biomimetics7030117 (PMC9496476; doi:10.3390/biomimetics7030117)
Supplement: Supplementary file 1 [file biomimetics-07-00117-s001.zip › biomimetics-1827521-supplementary.pdf]

Supplemental Information

Tailoring of TiAl6V4 Surface Nanostructure for Enhanced *in-vitro* Osteoblast Response via Gas/Solid (Non-Line-of-Sight) Oxidation/Reduction Reactions

Naotaka Ogura[1,#], Michael B. Berger[2,#], Pavan Srivas[1], Sunghwan Hwang[1], Jiaqi Li[1], D. Joshua Cohen[2], Zvi Schwartz[2,3,*], Barbara D. Boyan[2,4,*], and Kenneth H. Sandhage[1,*]

*Barbara D. Boyan
bboyan@vcu.edu
ORCID = 0000-0002-9642-0311

*Zvi Schwartz
zschwartz@vcu.edu
ORCID = 0000-0003-1612-9223

*Kenneth H. Sandhage
sandhage@purdue.edu
ORCID = 0000-0002-7957-1361

[1]School of Materials Engineering, Purdue University, W. Lafayette, IN 47907, USA
[2]Department of Biomedical Engineering, Virginia Commonwealth University, Richmond, VA 23284, USA
[3]Department of Periodontology, University of Texas Health Science Center at San Antonio, San Antonio, TX 78229, USA
[4]Wallace H. Coulter Department of Biomedical Engineering at the Georgia Institute of Technology and Emory University, Atlanta, GA 30332, USA

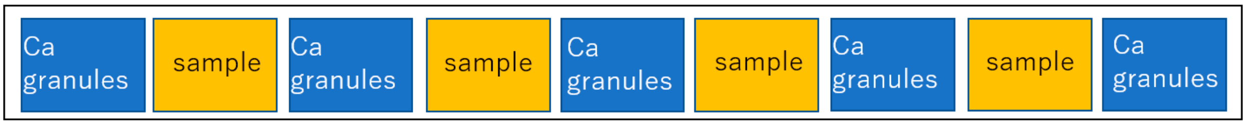

Supplemental Figure S1. Schematic illustration of the alternating arrangement of oxidized TiAl6V4 specimens (in Ti crucibles) with Ca granules (also in Ti crucibles), all of which were sealed within a steel ampoule, for the calciothermic reaction step.

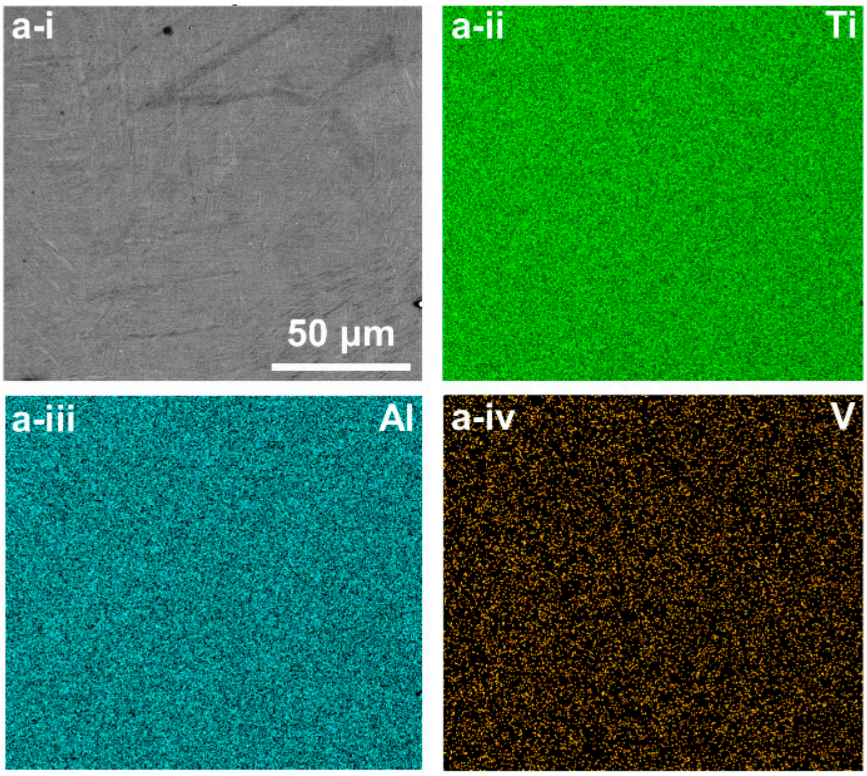

Supplemental Figure S2. Characterization of a starting polished DMLS TiAl6V4 specimen: **(a-i)** top-down BSE (backscattered electron) image of the surface and associated EDX elemental maps for **(a-ii)** titanium, **(a-iii)** aluminum, and (**a-iv)** vanadium.

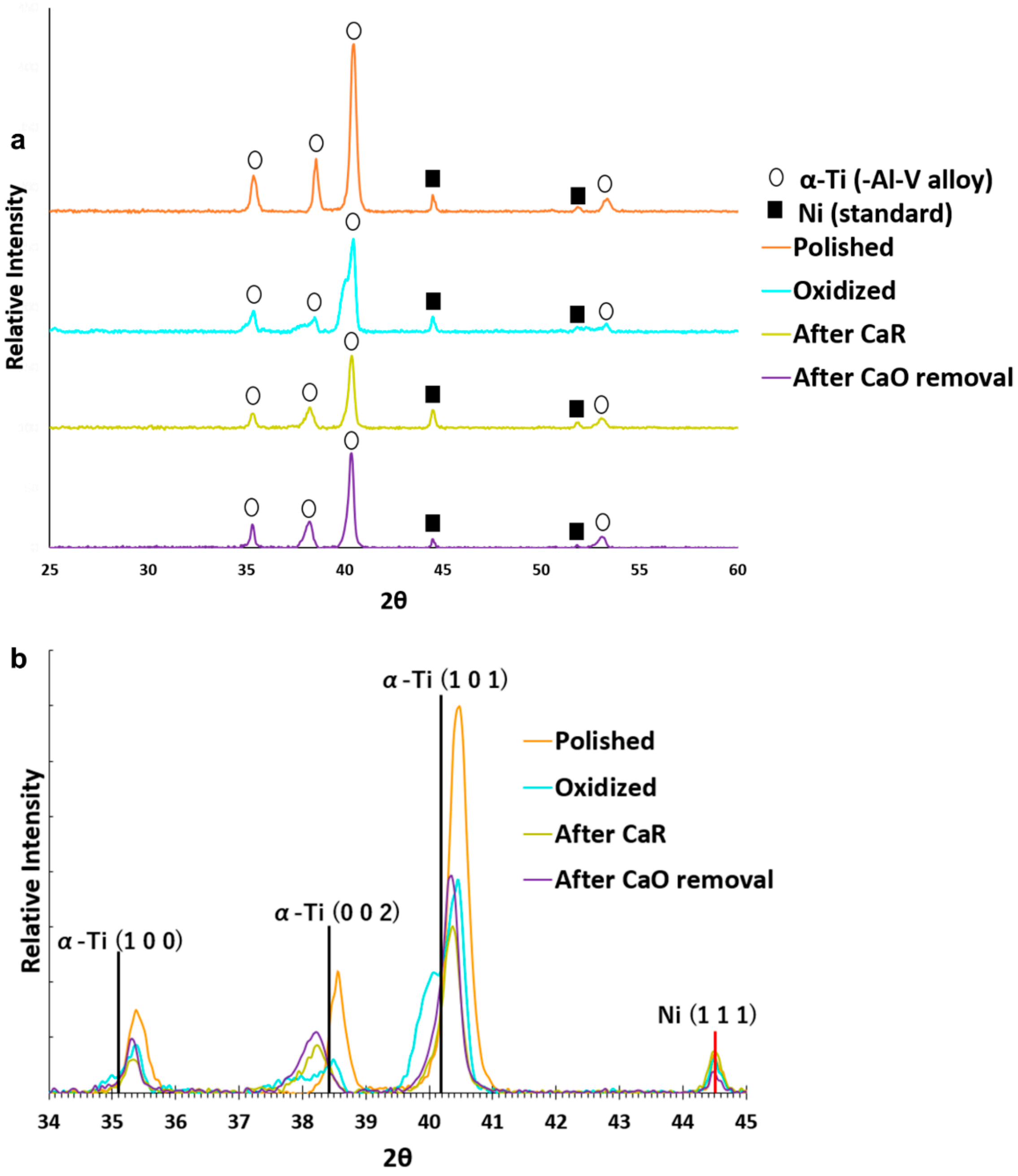

Supplemental Figure S3. XRD analyses of polished DMLS TiAl6V4 specimen surfaces after each reaction stage: (**a**) separated XRD patterns (2θ = 25-60 degrees); (**b**) superimposed XRD patterns (2θ = 34-45 degrees), with vertical black lines indicating the positions of diffraction peaks for pure α-titanium (note: "After CaR" indicates after the calciothermic reaction).

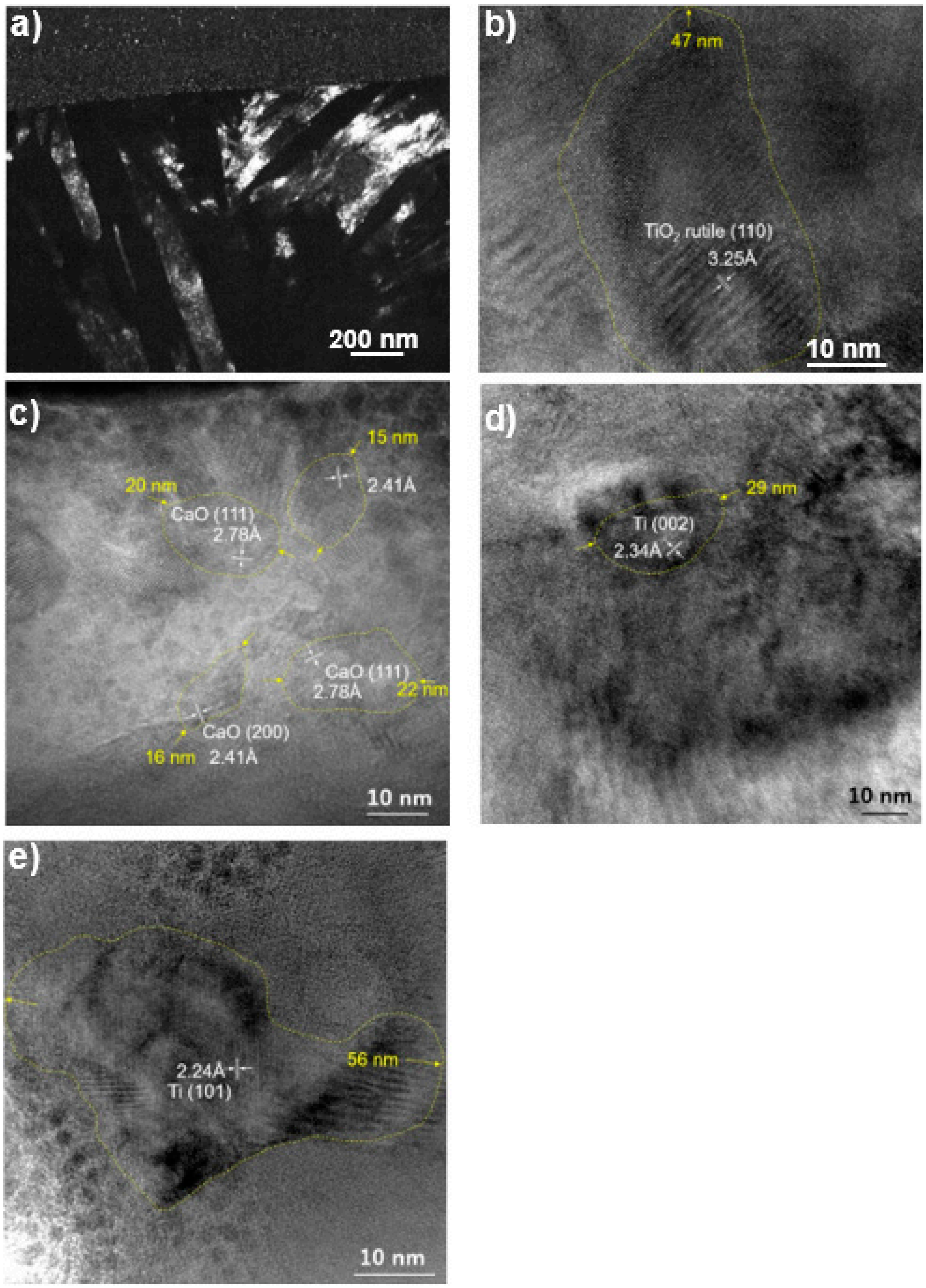

Supplemental Figure S4. TEM analyses at various stages of reaction of polished DMLS TiAl6V4 specimens: (**a**) Dark field TEM image of an ion-milled cross-section of the polished starting DMLS specimen revealing α-TiAl6V4 crystals; (**b**) HRTEM image of the nanocrystalline rutile TiO_2 layer generated upon oxidation at 600 °C for 6 h in air; (**c**) HRTEM image of the nanocrystalline CaO layer formed upon reaction of the oxidized TiAl6V4 specimen with Ca(g) at 700 °C for 1 h; (**d**) HRTEM image of the nanocrystalline α-Ti-rich layer formed upon reaction of the oxidized TiAl6V4 specimen with Ca(g) at 700 °C for 1 h; (**e**) HRTEM image of the nanocrystalline α-Ti-rich layer after selective acetic acid dissolution of the CaO generated upon reaction of the oxidized TiAl6V4 specimen with Ca(g) at 700 °C for 1 h.

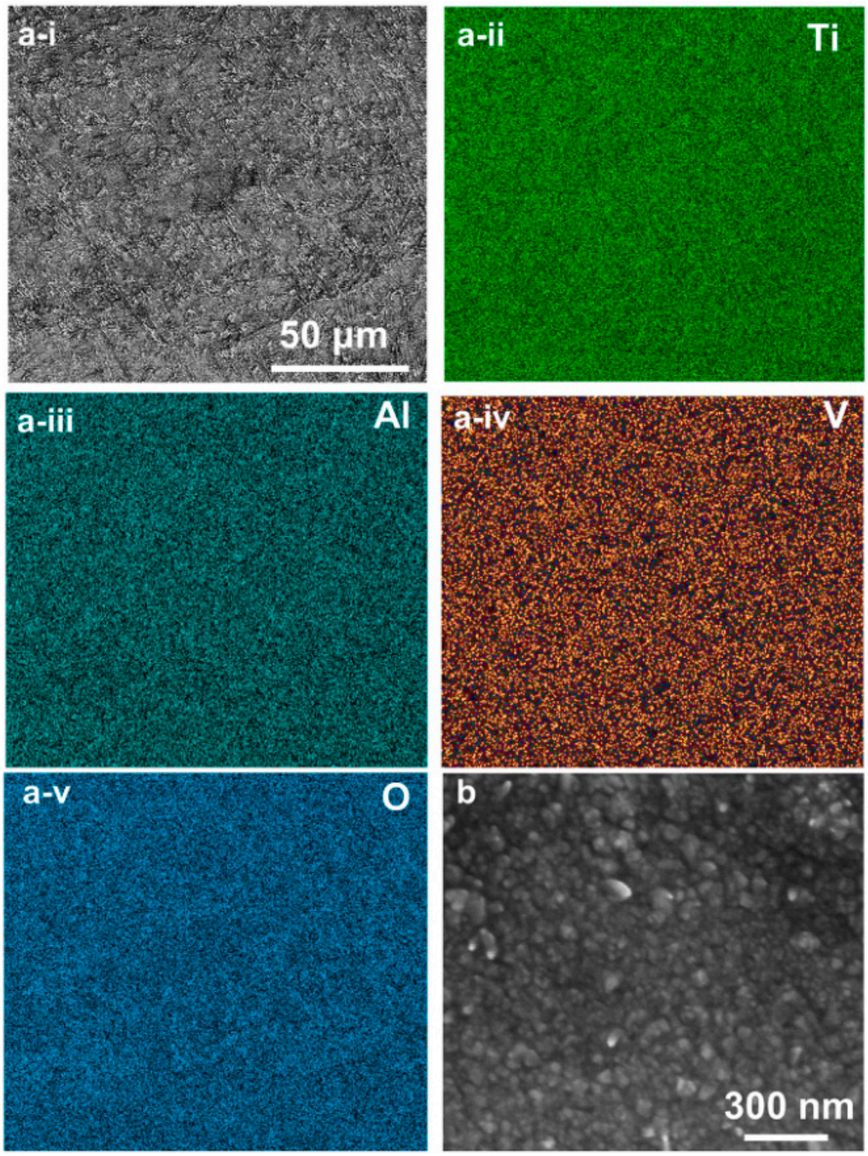

Supplemental Figure S5. Characterization of a polished DMLS TiAl6V4 specimen after exposure to air at 600ºC for 6 h: (**a-i)** top-down BSE image of the surface and associated EDX elemental maps for (**a-ii)** titanium, (**a-iii)** aluminum, (**a-iv)** vanadium, and (**a-v)** oxygen; (**b)** higher-magnification top-down SE image of the surface

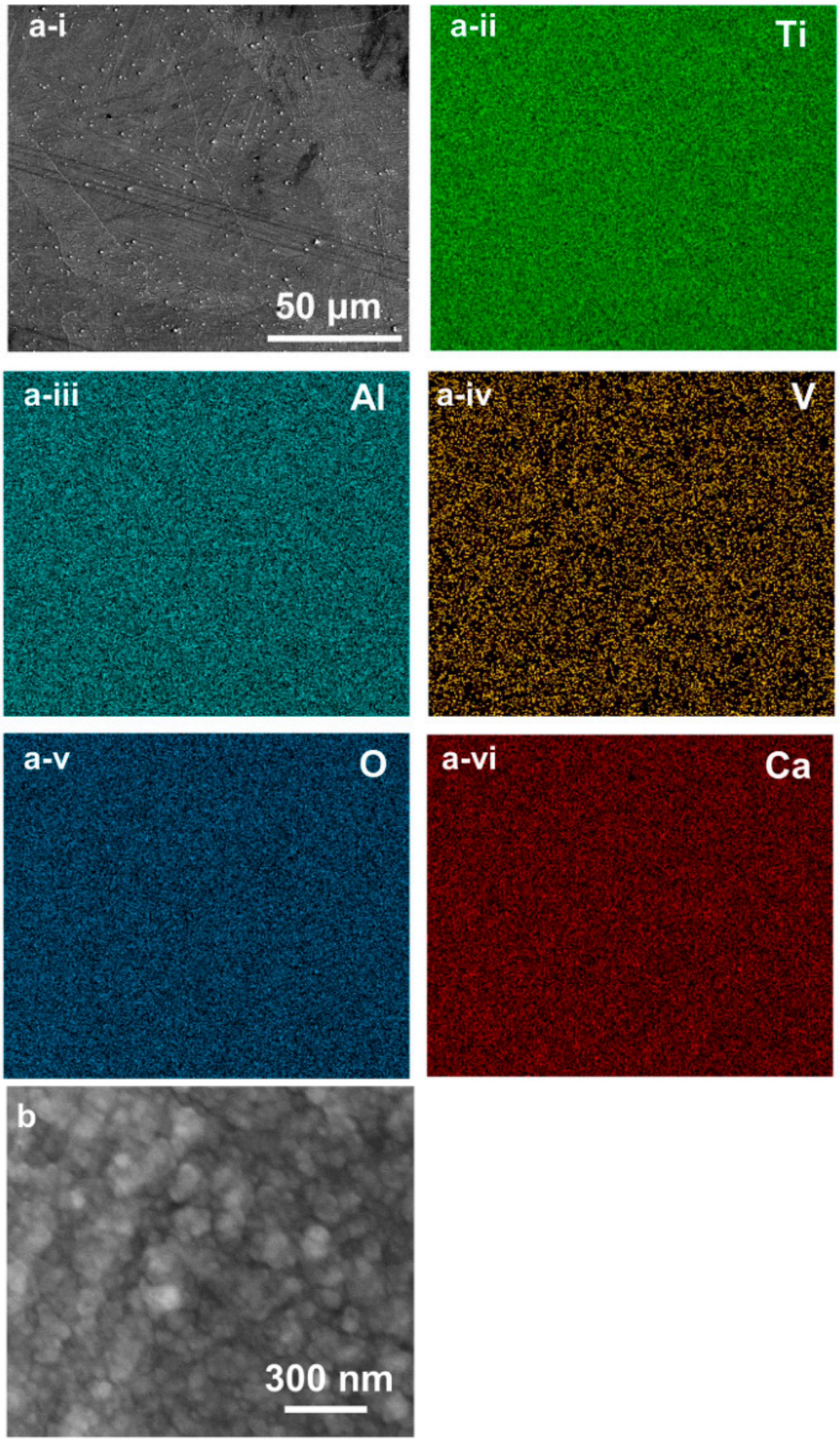

Supplemental Figure S6. Characterization of a polished, oxidized DMLS TiAl6V4 specimen after calciothermic reaction at 700ºC for 1 h: **(a-i)** top-down BSE image of the surface and associated EDX elemental maps for; **(a-ii)** titanium; **(a-iii)** aluminum; **(a-iv)** vanadium; **(a-v)** oxygen; and **(a-vi)** calcium; **(b)** higher-magnification top-down SE image of the oxidized surface.

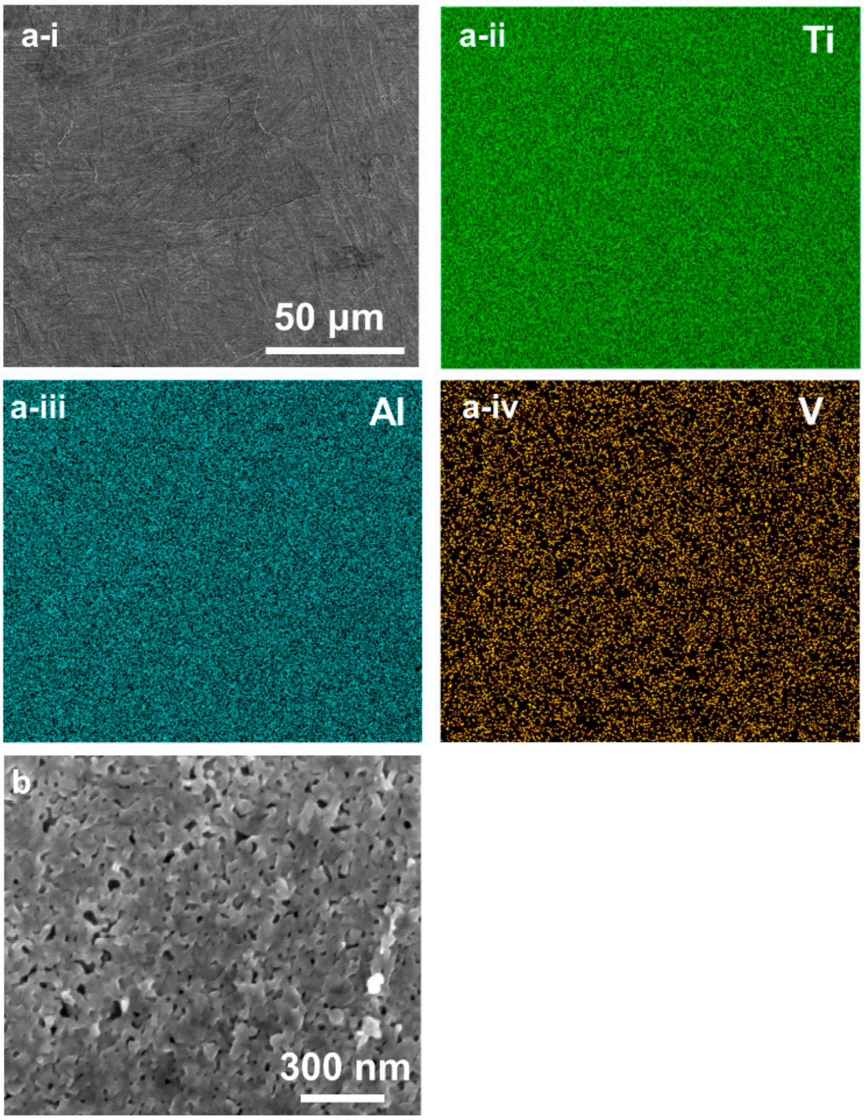

Supplemental Figure S7. Characterization of a polished, oxidized, and calciothermically-reacted DMLS TiAl6V4 specimen after selective CaO dissolution: **(a-i)** top-down SEM image of the surface and associated EDX elemental maps for **(a-ii)** titanium, **(a-iii)** aluminum, and **(a-iv)** vanadium; **(b)** Higher-magnification top-down SEM image of the surface.

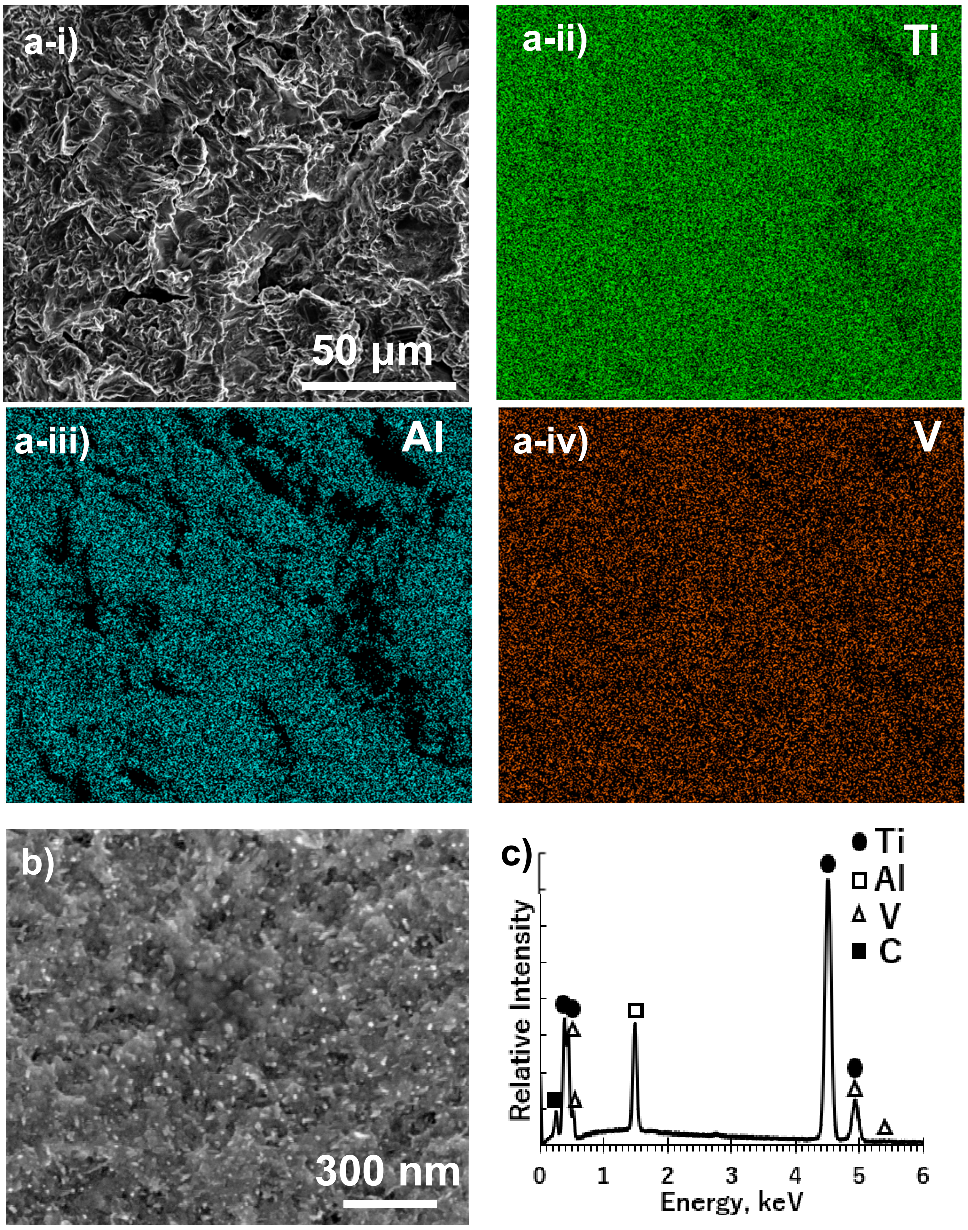

Supplemental Figure S8. Characterization of a starting microrough (grit-blasted and acid-etched) DMLS TiAl6V4 specimen: **(a-i)** SEM image of the external surface and associated EDX elemental maps for **(a-ii)** titanium, **(a-iii)** aluminum, and **(a-iv)** vanadium; **(b)** higher-magnification SEM image of the external surface; **(c)** SEM/EDX spectrum obtained from the external surface.

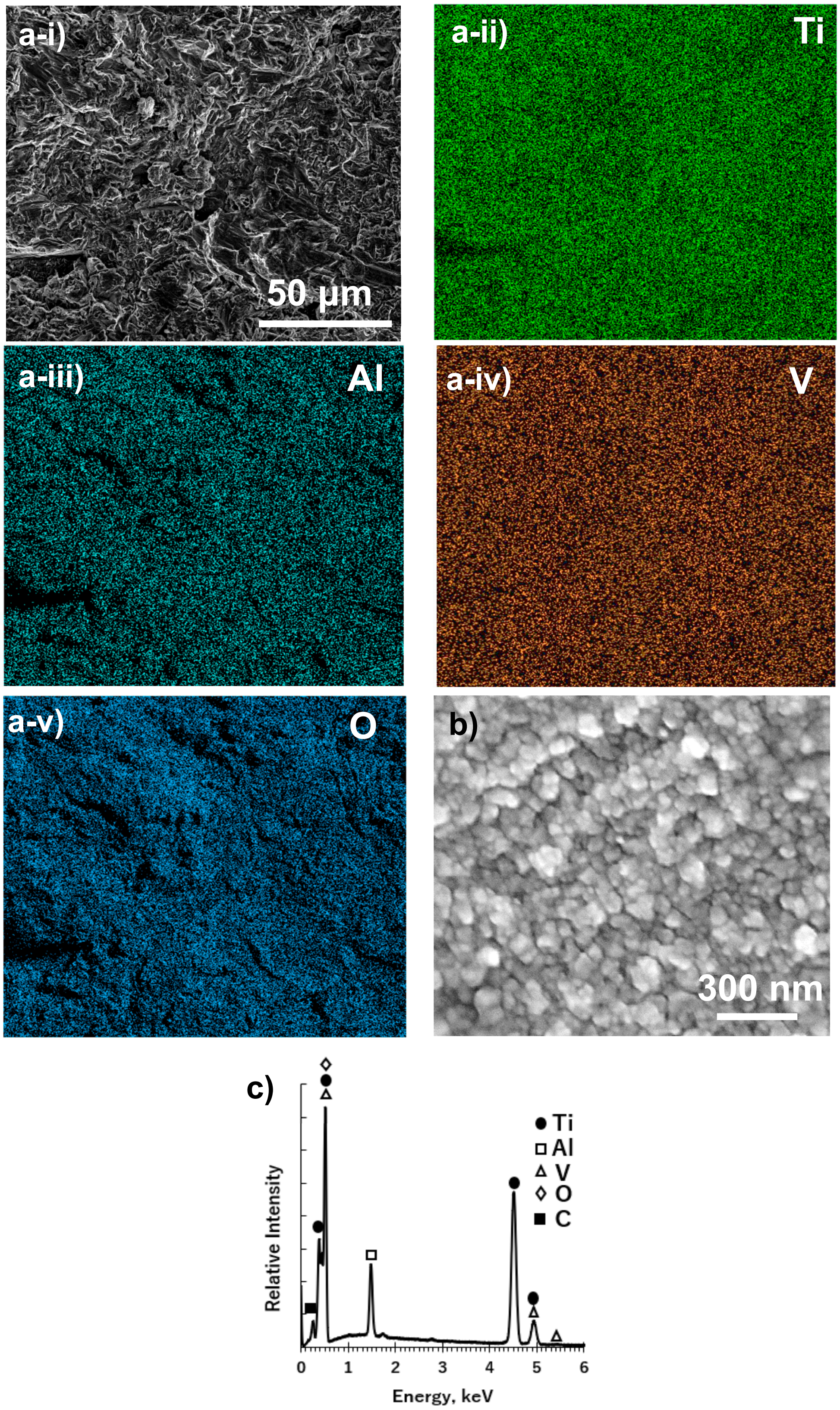

Supplemental Figure S9. Characterization of a microrough (grit-blasted and acid-etched) DMLS TiAl6V4 specimen after oxidation in air at 600°C for 6 h: (**a-i)** SEM image of the external surface and associated EDX elemental maps for (**a-ii)** titanium, (**a-iii)** aluminum, (**a-iv)** vanadium, and (**a-v)** oxygen; (**b)** higher-magnification SEM image of the external surface; (**c)** SEM/EDX spectrum obtained from the external surface.

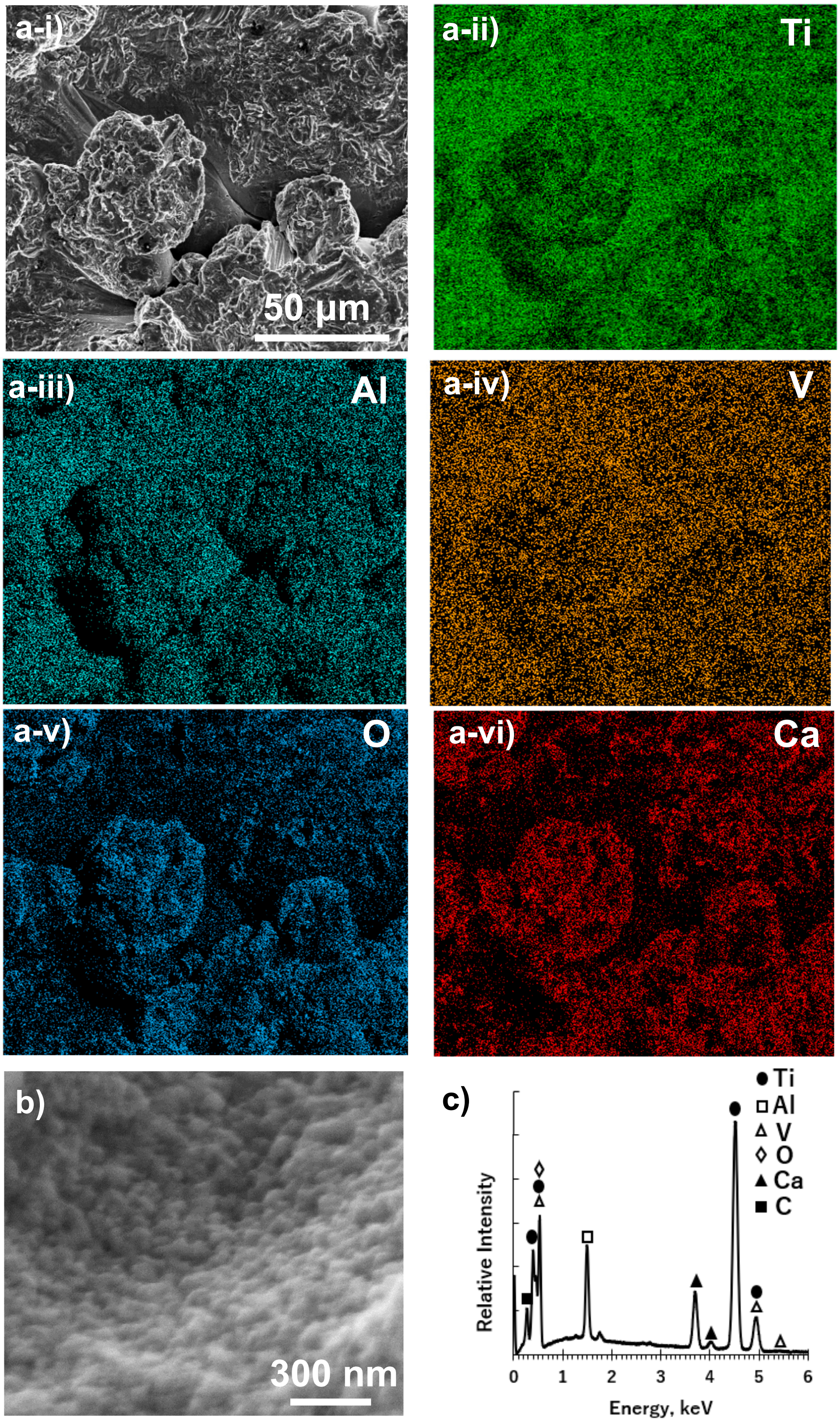

Supplemental Figure S10. Characterization of a microrough (grit-blasted and acid-etched), oxidized DMLS TiAl6V4 specimen after calciothermic reaction at 700 ºC for 1 h: (**a-i)** SEM image of the external surface and associated EDX elemental maps for (**a-ii**) titanium, (**a-iii**) aluminum, (**a-iv**) vanadium, (**a-v)** oxygen, and (**a-vi**) calcium; (**b**) higher-magnification SEM image of the external surface; (**c**) SEM/EDX spectrum obtained from the external surface.

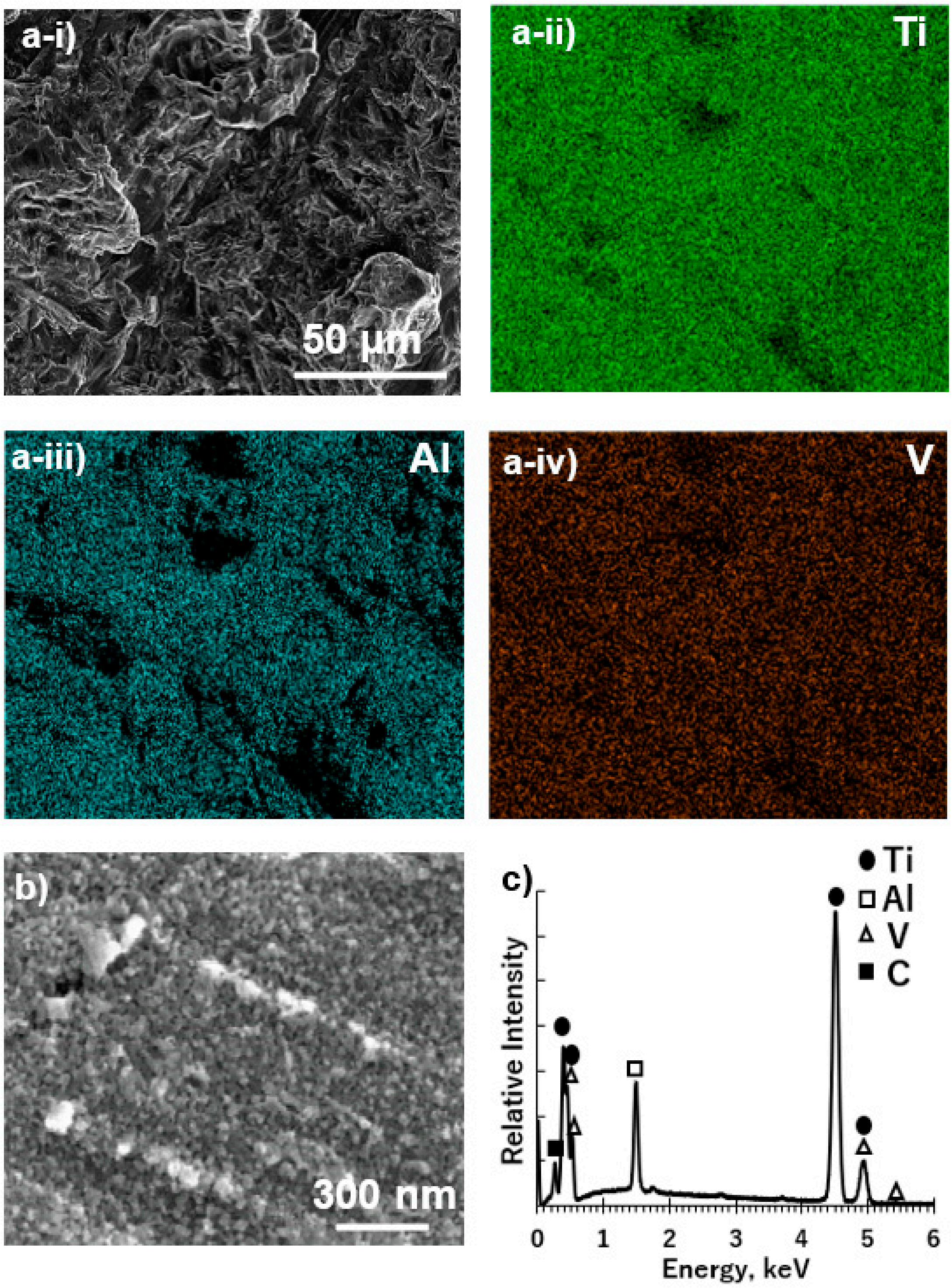

Supplemental Figure S11. Characterization of a microrough (grit-blasted and acid-etched), oxidized, calciothermically-reacted DMLS TiAl6V4 specimen after selective CaO dissolution: **(a-i)** SEM image of the external surface and associated EDX elemental maps for **(a-ii)** titanium, **(a-iii)** aluminum, and **(a-iv)** vanadium; (**b**) higher-magnification SEM image of the external surface; (**c**) SEM/EDX spectrum obtained from the external surface.

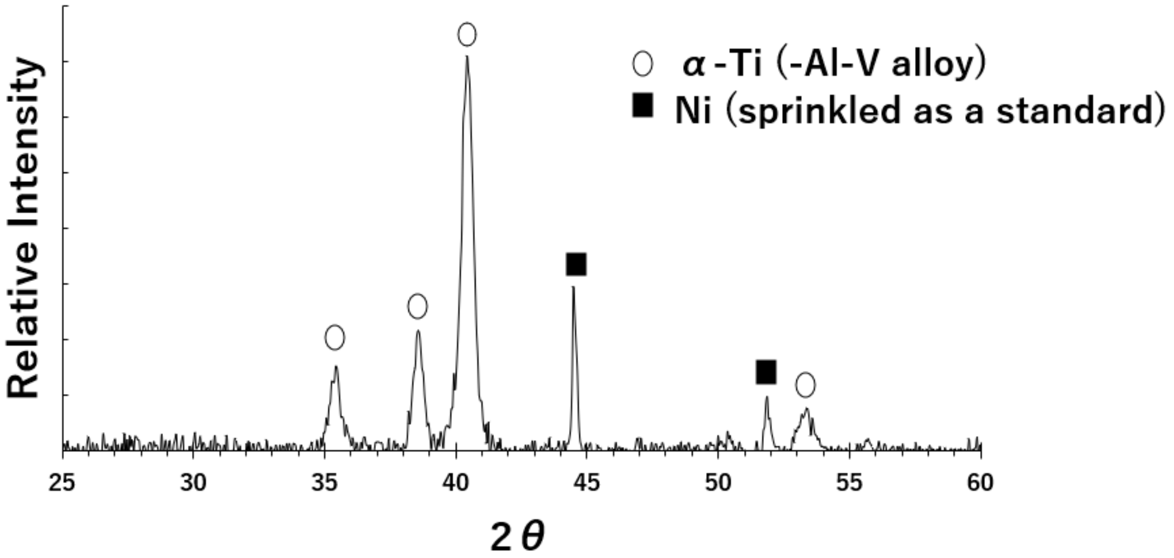

Supplemental Figure S12. XRD analyses of a starting microrough (grit-blasted, acid-etched) DMLS TiAl6V4 specimen.

Supplemental Table S1. Normalized compositions obtained from top-down SEM/EDX analyses of the surfaces of TiAl6V4 specimens at various stages of surface modification.

Specimen Condition	Ti (at%)	Al (at%)	V (at%)	O (at%)	Ca (at%)	Ti (wt%)	Al (wt%)	V (wt%)	O (wt%)	Ca (wt%)
As polished (Fig. 2b)	86	10	4	---	---	90	6	4	---	---
After oxidation (Fig. 3c)	38	5	2	55	---	63	4	3	30	---
After calciothermic reduction (Fig. 4c)	43	5	2	43	7	64	4	3	21	8
After CaO dissolution (Fig. 5c)	86	10	4	---	---	90.	6	4	---	---
